# Testing a novel multicomponent intervention to reduce meat consumption in young men

**DOI:** 10.1371/journal.pone.0204590

**Published:** 2018-10-11

**Authors:** Catherine E. Amiot, Guy El Hajj Boutros, Ksenia Sukhanova, Antony D. Karelis

**Affiliations:** 1 Department of Psychology, Université du Québec à Montréal, Montreal, Quebec, Canada; 2 Department of Exercise Science, Université du Québec à Montréal, Montreal, Quebec, Canada; UT School of Public Health, UNITED STATES

## Abstract

Both epidemiological studies and randomised controlled trials have shown that meat-eating can be harmful to human health. Meat-eating is also considered to be a moral issue, impacting negatively on the environment and the welfare of animals. To date, very little scientific research has aimed to reduce this dietary behavior. Therefore, the current research tests the effectiveness of a 4-week multicomponent intervention designed to reduce meat-eating. Using a randomised controlled trial procedure, thirty-two young men (mean age: 23.5 ± 3.1 years old) were randomly assigned into two equal groups, the intervention vs control group. Based on research in social and health psychology, the intervention was composed of five components expected to reduce meat consumption: a social norm component; an informational/educational component; an appeal to fear; a mind attribution induction; and a goal setting/self-monitoring component. Measures of different types of meat intake (using dietary journals) were taken at baseline (Time 1) as well as 2 (Time 2) and 4 weeks later (Time 3). Emotions and attitudes toward meat-eating and animals were also assessed at Time 3. Significant reductions in total and weekend red meat consumption as well as cold cuts consumed on the weekend were observed in the intervention condition from Time 1 to Time 3. Moreover, reduced positive emotions toward eating meat mediated the reduction in red meat consumption. The component of the intervention that participants most often perceived as having led to a reduction in their meat consumption was the informational component. In conclusion, results provide support for the effectiveness of the multicomponent intervention and for the mediating role of positive emotions when predicting behavioral changes in meat consumption.

## Introduction

The empirical evidence showing how meat-eating can be detrimental to human health is accumulating. Epidemiological and prospective studies have revealed that meat-eating is associated with a greater likelihood of developing certain diseases such as colorectal [1], gastric [2], or pancreatic cancer [3] as well as diabetes, stroke, coronary heart disease, and heart failure [4–5]. Randomised controlled trials have further shown that plant-based (compared to omnivore) diets may lead to better physiological and psychological well-being in terms of weight loss, lower dietary inflammation index (an indicator of diet quality) [6–7], decreased symptoms of osteoarthritis [8], and even better mood [9]. On the basis of these findings, the World Health Organization in 2015 recommended the consumption of red meat on an exceptional basis and to avoid processed meat altogether.

Meat-eating is also considered an ethical and moral issue [10], with meat-animals destined to live lives full of suffering and ending in a painful death [11]. In addition, workers in meat-processing facilities report high levels of mental and physical health problems [12]. Meat-eating has also been identified as a direct contributor of climate change given the resources and energy required to produce meat [13–14]. These environmental issues are now recognized as likely to have, in the medium- and longer-term, detrimental consequences on human health [15].

Overall, meat consumption in Canada remains high. In 2015, only 8% of surveyed Canadians identified themselves as vegetarians or mostly vegetarians [16]. Men also seem to be more accepting of meat consumption than women due to their beliefs that eating meat evokes masculinity, strength, and toughness [17]. However, 25% of surveyed participants in Canada indicated their desire to eat less meat, with young Canadians currently constituting the largest group of vegetarians [16]. Together, these trends point to a high potential for reducing meat consumption within the Canadian population in the next years, especially among younger groups (see also [18]). And considering men’s existing beliefs about meat, this segment of the Canadian population could potentially benefit the most from a meat-reduction intervention.

Given these facts, the current research tests the effectiveness of an innovative and integrative intervention that aims to reduce meat consumption. To this aim, we bring together procedures and notions from social and health psychology which are known to produce behavioral changes. Whereas the primary objective of this intervention was to modify actual meat-eating behaviors over time, we also investigated if the intervention affects participants’ attitudes and emotions toward meat-eating and animals. On the basis of social psychological theories [19], we specifically test if the impact of the intervention on actual meat consumption is explained (mediated) by changes in the attitudinal and emotional variables per se (see also [20]).

### Components of the intervention

Given the challenges associated with changing a habitual, enjoyable, and widespread behavior such as meat consumption [21–22], our intervention includes different components which, when combined, are expected to yield to significant changes in meat-eating behaviors, attitudes, and emotions (see also [23]).

The first component of our intervention builds on social psychological research pertaining to **social norms**, which are defined as general standards for behaviors and attitudes within a relevant social group [24]. Norms have been shown to affect and predict a wide range of individual behaviors, such as environmental behaviors [25] and eating habits [26], and intergroup behaviors, such as discrimination and fairness [27]. Although social norms that promote meat-eating are currently widespread in North America and that a wide majority of Americans are omnivores (i.e., 94%; [28]), these norms are now changing. Indeed, more and more Americans consider reducing their meat consumption [29]. In Canada, there has been a general drop in meat-eating from 1980 to 2015 observed in beef (37% reduction), pork (30% reduction), and veal consumption (37.5% reduction) [30]. Given that becoming aware of these emerging social norms could also encourage people to join this change effort, one component of our intervention will hence present these shifting norms [31].

The intervention also includes an **informational/educational approach**, mainly based on self-determination theory (SDT) [32]. According to SDT, when provided with a rationale and information for engaging in certain behaviors (or not), people are then more likely to be autonomous and persistent in the display of these behaviors [33]. This positive association between providing information/autonomy support and adopting more healthy behaviors has also been found in the realm of nutrition [34–35]. On that basis, presenting people with information about the reasons why it is important to reduce or avoid meat-eating and highlighting the benefits of doing so to one’s health is likely to produce changes in this specific behavior and associated attitudes. Providing such information is particularly important in the context of meat consumption given that one of the reasons for not reducing one’s meat consumption is the lack of knowledge about plant-based and meat-based diets, which results in a belief that meat is essential to health [36].

The intervention also encourages **goal setting and self-monitoring**, as two motivational elements that are frequently incorporated in interventions simultaneously (e.g., [37–40]), a combination which can be particularly powerful [41]. Asking participants to both set a goal with respect to their meat-eating and following-up with them about this behavior–using texting in the case of our intervention–should play a potent role in changing meat-eating behaviors and attitudes. Indeed, text-messaging has been found to be an effective method to promote behavioral changes in various contexts, from physical activity to asthma self-management [42] (see [43] for a meta-analysis).

**Appealing to fear** will also be used to reduce meat-eating behaviors. Research has found that the stronger the fear appeal, the greater its effects, especially when it is accompanied by concrete solutions on how to avoid the negative consequences of the behavior targeted [44]. Fear appeal has been successfully used to produce behavior change such as smoking cessation [45], cancer prevention behaviors [46], and responsible driving [47]. A meta-analysis [48] showed an overall positive effect of fear appeal for changing a variety of additional health-related behaviors and attitudes, including disease prevention and reduced drinking. However, threatening messages have been found to be more successful when people feel self-efficacious in changing their behaviors [49]; for this reason, it is important to supplement the fear-eliciting messages in the intervention with the presentation of valid strategies for behavior modification. The current intervention hence presents concrete solutions to deal with the problem of animal farming and harm.

In terms of **mind attribution**, Bastian, Loughnan, Haslam, and Radke [50] have found that animals who are raised for human consumption are typically denied mental capacities. This denial of mind to farm animals then allows individuals to decrease the internal tension and dissonance associated with enjoying eating meat and feeling compassion toward animals at the same time–i.e., the meat-paradox (see also [10]). Instead, activating mind attribution to meat-animals–a process that’s somewhat antithetical to mind denial as it involves attributing human-like states such as feelings, thoughts, and intentions to animals [51]–is likely to lower meat consumption and trigger less positive attitudes and emotions toward meat-eating.

### Prior and current research on reducing meat consumption

There is currently limited research on the best practices to reduce meat consumption. In one of the few studies on the topic, Allen and Baines [52] manipulated the symbolized meaning of meat-eating in an aim to change meat consumption. Participants were informed that meat-eating is an attribute of people who are in favor of social hierarchy–i.e., who endorse social dominance values. Results showed that, following this manipulation, participants who also rejected the ideology of social dominance reported more negative attitudes toward meat consumption. However, a follow-up assessment three weeks after the experiment showed that the manipulation did not produce actual behavioral changes in meat consumption per se. Therefore, the current study aimed to test the effectiveness of an intervention that should produce concrete behavioral effects on meat consumption.

In an online experiment conducted by Cordts, Nitzko, and Spiller [53], German participants were presented with one of four educational messages that aimed to modify their attitudes toward meat and meat-eating behaviors. These messages were about animal welfare (i.e., eating meat contributes to animal suffering), health (i.e., meat-eaters have lower life expectancy and higher risk to develop certain diseases), climate change (i.e., eating meat contributes to greenhouse gas emissions), and personal image (i.e., by portraying meat-eaters as less likeable among colleagues and friends). While each of these messages were effective in inducing the desire to eat less meat just following the manipulation (compared to a control condition), the message about animal welfare was found to be the most effective in promoting greater intentions to reduce meat consumption, suggesting that referring to animals may be one potent means to impact on health behaviors per se.

Stea and Pickering [54] also tested how the content of a message can affect people’s intentions to eat less meat. They varied the framing of six messages about the effects of meat production on the environment and randomly assigned participants to read one of these messages. The framing of the messages varied in terms of social norms around meat and salience of the Canadian identity. Importantly, including social norms aspects in the message produced the strongest effect. These findings suggest that it is possible to increase intentions to eat less meat by presenting informational messages. However, in Stea and Pickering’s study, one significant limitation is that participants’ actual meat eating behaviors were not assessed, just their intentions, making it impossible to know if their manipulations were successful in reducing meat consumption.

Carfora, Caso, and Conner [55] more recently conducted a randomised controlled trial to address this limitation and test the effectiveness of an intervention designed to produce changes in actual meat-eating behavior. They showed that a 7-day text-messaging intervention, which reminded participants to monitor their red meat consumption, was effective in increasing intentions to eat less red meat and in actually reducing this dietary behavior. Although the researchers obtained a significant behavioral effect, it remains to be tested if changes in meat consumption can be sustained over a longer timeframe, also beyond the time period during which the text messages are sent. Therefore, and in comparison to these prior studies, the present research contributes significantly to the existing literature in three important and novel ways: 1) We test the effectiveness of a multicomponent intervention on participants’ actual meat consumption (rather than just their behavioral intentions), 2) The research design employed examines these effects over a longer timeframe (i.e., a 4-week period, rather than 1 week as was done in some prior work), and 3) The current work directly captures the psychological processes (i.e., emotions) that account for these changes over time.

Specifically, and using a randomised controlled trial procedure, the current study tested the effectiveness of a novel intervention compared to a control condition over a total period of 4 weeks. Meat consumption was assessed at three time points during this period: At the start of the study (baseline; Time 1), 2 weeks later (Time 2), and 4 weeks later (Time 3). We expected significant reductions in meat consumption across these time points among the intervention group per se, and more specifically between Time 1 –as the baseline–and Time 2, and also between Time 1 and Time 3. The attitudinal and emotional variables were expected to mediate the association between the condition (intervention vs. control) and changes in meat consumption over time [20].

## Methods

### Participants

To establish the sample size required for our intervention, we relied on the prior intervention study that assessed actual changes in meat-eating behavior over one week [55]. In this study, the authors observed a small-medium effect size for their intervention (η^2^ = .12). On this basis, the G*Power software was used to calculate the sample size required to detect a small-medium effect size (*f*^2^ = .37) in an experiment with 2 groups and 3 measurements points (Time 1 = pre-test, Time 2 = 2 weeks later, Time 3 = 4 weeks later) with a level of power of (1—*β*) >.95 (a conservative estimate also given the longer timeframe of this study) and *α* = .05. To take into account potential drop-outs from the intervention, 5 participants were added to each condition.

A total of thirty-two male participants were hence recruited for this 4-week randomised controlled trial study using advertisements via emails, social media, and presentations in classrooms at Université du Québec à Montréal. Participants were included in the study if they met the following criteria: 1) male 2) aged between 18–30 years old, 3) a body mass index (BMI) between 18.5–29.9 kg/m^2^, 4) omnivore (eating meat at least 3–4 times per week), 5) francophone and born in the Province of Quebec, 6) Caucasian, and 7) possessed a cellular phone. Exclusion criteria were: 1) chronic diseases such as cardiovascular disease, diabetes, cancer, 2) eating disorders, 3) currently following a weight loss program, and 4) currently possessing a pet at home, given the association between pet keeping and positive attitudes toward animals [56], including meat-animals [17]. The criterion of consuming meat at least 3–4 times per week allows to prevent against observing floor effects by including only those participants who currently consume a significant amount of meat. In addition, eating meat 3–4 times a week has been found to be a prevalent frequency among meat-eaters [54].

The present population was selected in order to ensure homogeneity within the sample. Furthermore, men have been shown to eat more meat than women [57–58], to have slightly lower positive attitudes toward animals, and to be less likely to be animal right activists [59]. Eating meat has also been associated with perceptions of masculinity and strength [60–61]. If our results are conclusive for male participants, this would provide encouraging evidence for the effectiveness of our intervention. Young adults (18–30 years-old) were also recruited for the study given that they are in a period where they are constructing and developing their eating habits [62]. Changing their habits at this specific period of life could hence be beneficial over the longer-term. The study was conducted in accordance with the Declaration of Helsinki and all procedures were approved by the Ethics Committee of the Université du Québec à Montréal. All participants were fully informed about the nature, goal, procedures and risks of the study, and gave their informed consent in writing. However, the goal of the experiment was presented as an overall investigation of eating habits rather than meat consumption in particular in order to prevent participants from modifying their behavior.

### Design and procedure

The study was conducted between the end of May and the end of June 2017. A trained research assistant was in charge of recruiting participants, conducting the sessions with all participants, and communicating with them throughout the different steps of the study. The study took place over four weeks and was comprised of six steps, with three in-lab sessions for the intervention group and two for the control group. All materials used were in French; the measuring instruments were translated into French by bilingual researchers.

#### Step 1

All participants came to the laboratory to provide demographic information, which included: name, age, country of origin, how much time the participant had resided in Canada (if not born in Canada), native language, ethnicity, university major, year of studies, average number of meat portions consumed per week, whether the participant normally ate alone or with someone else, number of hours exercising per week, number of hours spent at home per day, whether the participant had a pet and how many, how much time the participant spent with the pet, whether the participant followed a religion and which, political beliefs on social issues (on a scale from 1 = *liberal* to 7 = *conservative*), and socio-economic status (measured with a ladder, where 1 = *bottom* and 9 = *top*). Their weight and height were also taken.

Using a draw, participants were randomly assigned to the control or intervention group. They then received information about how to complete the dietary journals. Specifically, participants were instructed to keep a record of their food intake, including condiments and beverages, over two weekdays and one weekend day. Participants were instructed to write as much information as possible about the foods that they consumed (e.g., brand names, how the food was cooked). An example of a detailed meal and how to report it in the dietary journal was also provided to participants. Food weight scales were distributed to each participant in order for them to weigh their portions, in particular all meats. Participants were also asked to use the usual tools to estimate their portion sizes (i.e., teaspoon/tablespoon/cup in ml or ounces). On their return, each food record was reviewed in order to verify the precision of the information written. The dietary analyses of different types of meat–namely red meat, white meat, fish, and cold cuts during the week and weekend–were reported in grams. Each portion of meat was classified exclusively into one of these categories. Examples of red meat include burger patties and steak; examples of white meat include pork and poultry; examples of fish include salmon and canned tuna; examples of cold cuts include salami and ham. The first dietary journal was completed in the following week and represented a baseline measure of meat-eating.

#### Step 2 (intervention group only)

One week following Step 1, participants returned to the laboratory to take part in a one-on-one information session and return their first dietary journal. The information session involved a PowerPoint presentation delivered by the research assistant. This presentation first described emerging social norms that show a significant reduction in meat-eating since 1980 in Canada [30]. Participants were then presented with information about the negative effects of meat-eating, especially red and processed meats, on human health, animal welfare, and the environment. The presentation of such information allowed us to provide reasons and a rationale for reducing meat consumption [32]. Then, in the mind attribution task, participants were asked to describe a cow in a picture–as a typical farm animal–and to write a paragraph about the cow’s inner thoughts, feelings, intentions, and emotions (see [51]). In order to appeal to fear, two videos created by PETA (People for Ethical Treatment of Animals) about the negative treatment of meat-animals were incorporated in the presentation (http://www.peta.org/videos/meet-your-meat/; https://www.youtube.com/watch?v=mySePIgnfIs). We obtained permission from PETA to use these videos as part of our research.

To maximise the effect of this fear appeal and increase self-efficacy, participants then received concrete tips on what they can do to deal with this problem. Specifically, they were provided with various tips for planning meat-free meals, substituting meat products, and choosing meatless meals in restaurants. At the end of the information session, participants set a goal for the following month regarding their meat consumption by choosing one of the three following options: reduce the quantity of meat they consume, stay at the same level of meat consumption, or increase meat consumption. If they planned on changing their meat consumption in the next month, they were also asked to indicate by how much, by choosing one of the following four options: eat 1–3 less portions per week, eat 4–6 less portions per week, avoid eating meat altogether, or ‘other’.

#### Step 3 (intervention group only)

We followed-up with the intervention participants using text messaging during the subsequent two weeks. This procedure reinforced the informational and self-monitoring components of our intervention. The messages sent upbeat and educational information that reminded participants of the benefits of reducing meat consumption and provided them with additional tips and links to recipes (e.g., http://www.meatlessmonday.com/recipes/grilled-teriyaki-tofu-vegetable-shish-kebabs/; http://www.meatlessmonday.com/recipes/supreme-crispy-quinoa-vegetable-burgers/). The messages were sent out daily at approximately 5pm given that dinner typically contains more meat products than the other meals of the day (e.g., [63–64]). The messages sent differed on each day, but all participants received the same message on the same day.

#### Steps 4 and 5

At the end of week 2, all participants reported their meat consumption using a second dietary journal. They also completed a third dietary journal at the end of week 4; indeed, there is evidence to suggest that meat consumption is a habit that is likely to change gradually [65]. Importantly, doing so also allows to test if possible changes in meat consumption continue to take place even once participants have stopped receiving text messages (i.e., two weeks later).

#### Step 6

All participants were invited to the laboratory for the final session during which they returned the second and the third dietary journals and the food scales that had been provided to them. They also completed the following measures (described below) aimed to assess their attitudes and emotions toward meat and animals: self-determined motivation to eat meat, ambivalence toward meat-eating, positive and negative feelings toward eating meat, intraindividual conflict related to meat-eating behavior, and inclusion of animals in the self. Participants also reported their self-categorization in terms of meat consumption (i.e., omnivore, vegetarian, vegan, flexitarian). Participants in the intervention condition were specifically asked to indicate whether they had achieved the goal they had set at Step 2 and to identify the components of the intervention that were most effective for them in reducing meat consumption. Lastly, all participants provided their comments about the study and indicated what was the goal of the study in their opinion.

### Attitudinal and emotional measures

The measure of *self-determined motivation* to eat meat was based on the SDT motivational continuum. The items for this scale were adapted from Guay and colleagues [66] and reworded to fit the context of meat-eating. It consisted of eight items measuring intrinsic motivation (“Because I derive pleasure from eating meat”), integrated regulation (“Because eating meat is part of my lifestyle”), identified regulation (“Because I think it is important to eat meat”), introjected regulation (“Because I would feel bad if I didn’t eat meat”; “Because I feel that I have to eat meat to feel good about myself”), external regulation (“Because other people close to me insist that I eat meat”; “Because I do not want to disappoint the people I eat with”), and amotivation (“I don’t know; I really wonder why I even eat meat”). The items were measured on a 1(*does not correspond at all*) to 7(*corresponds exactly*) scale. First, we averaged the scores for the two items measuring the introjected and external regulations. Second, we computed the index of self-determined motivation as a difference between the self-determined motivations (i.e., intrinsic, integrated, identified) and the non-self-determined motivations (i.e., introjected, external, and amotivation). The resulted index of self-determined motivation varied between -3.50 to 16.50, with higher scores representing greater self-determined motivation to eat meat.

*Ambivalence toward eating meat* (α = 0.83) was measured with the scale developed by Berndsen and van der Pligt [67]. This scale consists of three items measuring attitudes toward meat-eating. Participants indicated the extent to which they feel conflict with regards to eating meat on a scale from 1(*I feel no conflict at all*) to 10(*I feel maximum conflict*); the extent to which they feel indecision about meat-eating on a scale from 1(*I feel no indecision at all*) to 10(*I feel maximum indecision*); and the reactions they have toward meat on a scale from 1(*I have completely clear reactions*) to 10(*I have mixed reactions*). Greater scores on this measure indicated greater ambivalence toward meat-eating.

Participants then completed a scale that measures *positive and negative feelings toward eating meat*. The items for this scale were developed by Desmet and Schifferstein [68]. Eleven items referred to positive emotions (e.g., satisfaction, enjoyment; α = 0.88), and 11 items referred to negative emotions (e.g., shame, anger; α = 0.84) experienced when thinking about eating meat. Results were analysed separately for positive and negative emotions. Higher scores indicated greater presence of positive or negative emotions.

The notion of *intraindividual conflict* applied to meat-eating (α = 0.89) refers to participants’ feeling of incoherence between their values and the practice of meat-eating. To assess this construct, we adapted five items from Amiot, Louis, Bourdeau, and Maalouf [69] to the context of meat-eating: e.g., “I feel a conflict between eating meat and my personal values”. Higher scores indicated greater feeling of intraindividual conflict for eating meat.

The final measure, *inclusion of animals in the self*, was based on an existing measure that assesses Inclusion of the Other in the Self [56]. This pictorial scale consists of seven Venn diagrams that vary in the degree of overlap between the circles that represent the self and animals. Each diagram is assigned a score from 1(*least overlapping*) to 7(*most overlapping*). The participants were prompted that the presented diagrams illustrated the relationship they have with animals and asked to select one illustration that best describes this relationship. Greater score indicates greater feeling of closeness toward animals in general.

## Results

All reported statistical analyses were conducted using SPSS 25. Participants were on average 23.50 years old (*SD* = 3.14; range: 19–30) and were all University students. At baseline, they consumed on average 10.50 portions of meat per week (*SD* = 4.26), performed 7.0 hours of exercise a week (*SD* = 4.39), and spent 5.0 hours a day at home (*SD* = 1.76). Out of 32 participants, 9.4% followed a religion. In addition, 75% of participants reported working an average of 27.1 work hours per week (*SD* = 12.35). Average body mass index (BMI), computed as weight in kilograms divided by squared height in meters, was 23.3 kg/m^2^ (*SD* = 1.98; range: 20.8–28.5). Finally, participants were mostly liberal in their political beliefs on social issues (*M* = 1.41, *SD* = 0.67; on a 1 = *liberal* to 7 = *conservative* scale) and reported above-average socio-economic status (*M* = 6.88, *SD* = 1.01), measured with a socio-economic ladder using a scale from 1 (*bottom*) to 9 (*top*). Table 1 presents the distribution of sociodemographic variables across the two groups. As can be seen in this Table, no statistically significant differences were found between the groups on these variables.

**Table 1:** Distribution of sociodemographic variables by experimental condition; *N* (%).

Variable	Intervention *n* = 16	Control *n* = 16	Statistic
**Age**			***F* (1,30) = 0.05**
*M* (*SD*)	23.38 (3.34)	23.63 (3.03)	
**Country of origin**			**χ**^**2**^ **(3) = 4.17**
Canada	5 (31.25%)	8 (50%)	
Belgium	0 (0%)	2 (12.50%)	
France	9 (46.26%)	5 (31.25%)	
Lebanon	2 (12.50%)	1 (6.25%)	
**Native language**			
French	16 (100%)	16 (100%)	-
**Meat portions per week**			***F* (1,30) = 1.27**
*M* (*SD*)	11.38 (3.63)	9.69 (4.77)	
**Do you eat alone or in company of others?**			**χ**^**2**^ **(1) = 0.50**
Alone	7 (43.75%)	9 (56.25%)	
In the company of others	9 (56.25%)	7 (43.75%)	
**Year of Studies**			**χ**^**2**^ **(6) = 8.93**
First	2 (12.5%)	1 (6.25%)	
Second	6 (37.5%)	6 (37.5%)	
Third	7 (43.75%)	3 (18.75%)	
Fifth	0 (0%)	1 (6.25%)	
Second (Master’s)	0 (0%)	1 (6.25%)	
Finished	1 (6.25%)	0 (0%)	
Missing	0 (%)	4 (16%)	
**Hours of exercise per week**			***F* (1,30) = 1.99**
*M* (*SD*)	8.09 (5.70)	5.93 (2.21)	
**Hours spent at home per day**			***F* (1,30) = 0.04**
*M*(*SD*)	4.94 (1.69)	5.06 (1.88)	
**Do you follow a religion?**			**χ**^**2**^ **(1) = 0.37**
Yes	1 (6.25%)	2 (12.50%)	
No	15 (93.75%)	14 (87.50%)	
**Political beliefs**			***F* (1,29) = 0.83**
*M* (*SD*)	1.31 (0.60)	1.53 (0.74)	
**Do you work?**			**χ**^**2**^ **(1) = 0.67**
Yes	13 (81.25%)	11 (68.75%)	
No	3 (18.75%)	5 (31.25%)	
**Hours of work per week**			***F* (1,22) = 0.03**
*M* (*SD*)	27.46 (12.70)	26.64 (12.54)	
**Socio-Economic Status**			***F* (1,30) = 2.03**
*M* (*SD*)	6.63 (1.15)	7.13 (0.81)	
**Body Mass Index**			***F* (1,30) = 0.29**
*M* (*SD*)	23.53 (2.19)	23.15 (1.80)	

*Note*. All statistics are below the significance level of 0.05.

To ensure that the intervention and control groups did not differ on the baseline meat consumption measures, we conducted one-way ANOVAs with the intervention condition as an independent variable. No differences were observed at baseline between the groups for all meat consumption measures (all *F*s<2.02, *p*s>0.05, η^2^_p_s<0.07). Furthermore, none of the participants dropped out from the study over the 4-week period.

### Changes in meat consumption over time

To examine if meat consumption (in grams) differed across the intervention and control groups over the three time points, we first conducted a series of mixed ANOVAs with time as a within-participant factor and condition (intervention vs. control) as a between-participant factor. Given that relying solely on *p*-values to imply significance has been recently criticized [70], we elected to explore and interpret the effects observed in these ANOVAs by using the effect size of η^2^_p_ = 0.04 or higher as the lower cut-off. This specific cut-off represents the minimum effect size required to imply practical significance and to consider an effect as meaningful in social sciences [71]. Applying this criterion to the main analyses allowed us to be less conservative and also explore the effects we had hypothesized a priori.

Then, the meaningful and/or statistically significant interactions to emerge in the ANOVAs were interpreted by relying on multiple pairwise comparisons. Given our a priori interest in testing if meat consumption is reduced in the intervention group (but not in the control group) both from Time 1 to Time 2, and from Time 1 to Time 3, we hence focused on two specific comparisons: i.e., between Time 1 and Time 2, and between Time 1 and Time 3. In these comparisons, we also employed a Bonferroni correction to correct for this number of comparisons (i.e., 2) by lowering the significance threshold from *p* < 0.05 to *p* < 0.025.

**Fig 1:**
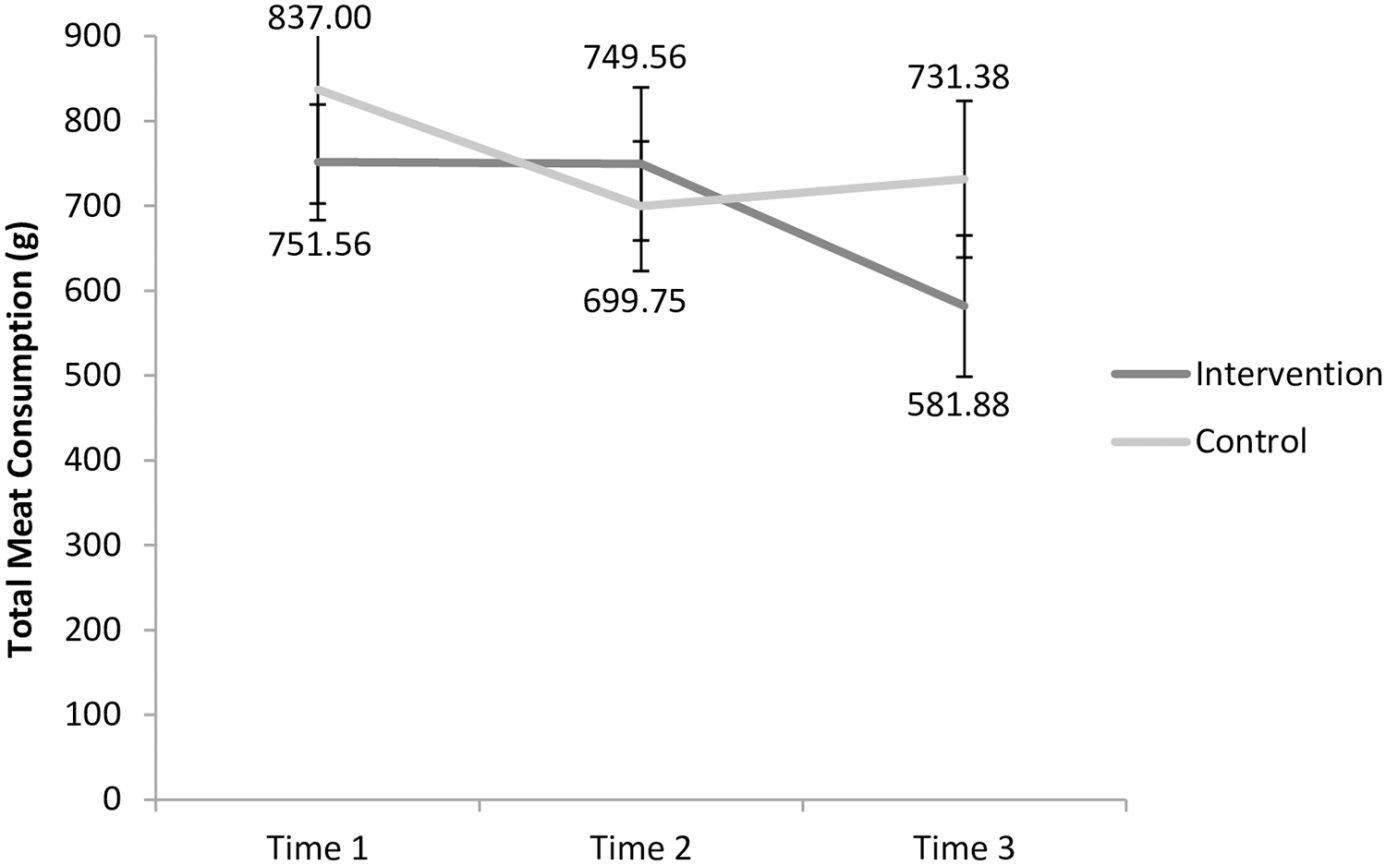
Changes in total meat consumption over time in the control and intervention groups. Error bars are based on the standard error of the mean.

As seen in Table 2, the ANOVA conducted on **total meat consumption** revealed an effect size that exceeded the η^2^_p_ = 0.04 threshold for the interaction between time and experimental condition. Fig 1 illustrates this interaction. The pairwise comparisons then conducted to interpret this interaction revealed that none of the individual contrasts within the control or the intervention group achieved the significance level of *p* = 0.025. While this analysis also revealed that the Time main effect exceeded the η^2^_p_ = 0.04 threshold, this main effect is interpreted in light of the interaction.

**Table 2:** Comparison of the control and intervention groups over time on meat consumption (in grams).

Variable	Intervention			Control			*F*_time_ (η^2^_p)_	*F*_cond_ (η^2^_p)_	*F*_int_ (η^2^_p)_
	Time 1 *M*(*SD*)	Time 2 *M*(*SD*)	Time 3 *M*(*SD*)	Time 1 *M*(*SD*)	Time 2 *M*(*SD*)	Time 3 *M*(*SD*)			
Total Meat Consumption	751.56 (272.47)	749.56 (360.97)	581.88 (332.55)	837.00 (535.43)	699.75 (305.00)	731.38 (369.51)	2.14 (.08)	0.33 (.01)	1.17 (.04)
Total Red Meat Consumption	315.13 (121.67)	291.00 (255.53)	129.06 (144.72)	418.63 (298.86)	360.06 (239.38)	363.81 (354.44)	3.11^†^ (.09)	4.04^†^ (.12)	1.59 (.05)
Total White Meat Consumption	314.06 (219.96)	289.50 (222.97)	241.44 (205.15)	293.75 (293.70)	194.50 (157.01)	193.69 (228.70)	1.81 (.06)	0.87 (.03)	0.33 (.01)
Total Fish Consumption	122.38 (132.11)	169.06 (231.95)	211.37 (358.97)	124.63 (144.52)	145.19 (144.77)	173.88 (199.83)	0.85 (.03)	0.20 (.01)	0.07 (.00)
Total Cold Cuts Consumption	156.13 (162.40)	150.00 (223.35)	90.06 (121.90)	135.50 (164.33)	110.19 (158.87)	120.06 (131.39)	1.03 (.03)	0.05 (.00)	0.79 (.03)
Red Meat Consumption during the Week	200.06 (113.03)	159.75 (139.33)	88.31 (118.25)	227.44 (219.75)	251.19 (204.93)	191.56 (251.39)	2.30 (.07)	2.42 (.08)	0.59 (.02)
White Meat Consumption during the Week	231.19 (192.60)	241.06 (182.33)	164.81 (145.68)	194.06 (221.52)	127.81 (131.62)	147.63 (203.97)	0.88 (.03)	1.02 (.03)	1.16 (.04)
Fish Consumption during the Week	73.69 (105.38)	119.38 (180.09)	149.81 (269.70)	104.31 (134.98)	71.25 (92.67)	146.81 (199.24)	1.05 (.03)	0.04 (.00)	0.39 (.01)
Cold Cuts Consumption during the Week	112.06 (122.72)	72.19 (168.28)	61.81 (105.77)	71.13 (94.86)	82.00 (122.94)	48.69 (52.20)	1.02 (.03)	0.27 (.01)	0.49 (.02)
Red Meat Consumption on the Weekend	115.06 (106.97)	131.25 (142.57)	40.75 (70.23)	191.19 (186.40)	94.50 (98.16)	172.25 (193.35)	1.50 (.05)	2.44 (.08)	4.32* (.13)
White Meat Consumption on the Weekend	100.88 (106.15)	48.44 (78.61)	76.63 (98.82)	99.69 (148.99)	66.69 (102.34)	46.06 (89.17)	1.61 (.05)	.04 (.00)	0.44 (.01)
Fish Consumption on the Weekend	48.69 (92.78)	49.69 (91.73)	61.56 (107.34)	20.31 (55.69)	73.94 (120.37)	27.06 (54.34)	0.98 (.03)	0.39 (.01)	1.33 (.04)
Cold Cuts Consumption on the Weekend	44.06 (67.31)	77.81 (94.25)	28.25 (51.51)	64.38 (113.31)	28.19 (50.86)	71.38 (109.57)	0.04 (.00)	0.04 (.00)	3.98* (.12)

*Notes*. Time 1 indicates meat consumption three days prior to the intervention (baseline); Time 2 indicates meat consumption upon the completion of the intervention (2 weeks later); Time 3 indicates meat consumption two weeks after the completion of the intervention (4 weeks later).

^†^*p*<0.10;

*p<0.05.

**Fig 2:**
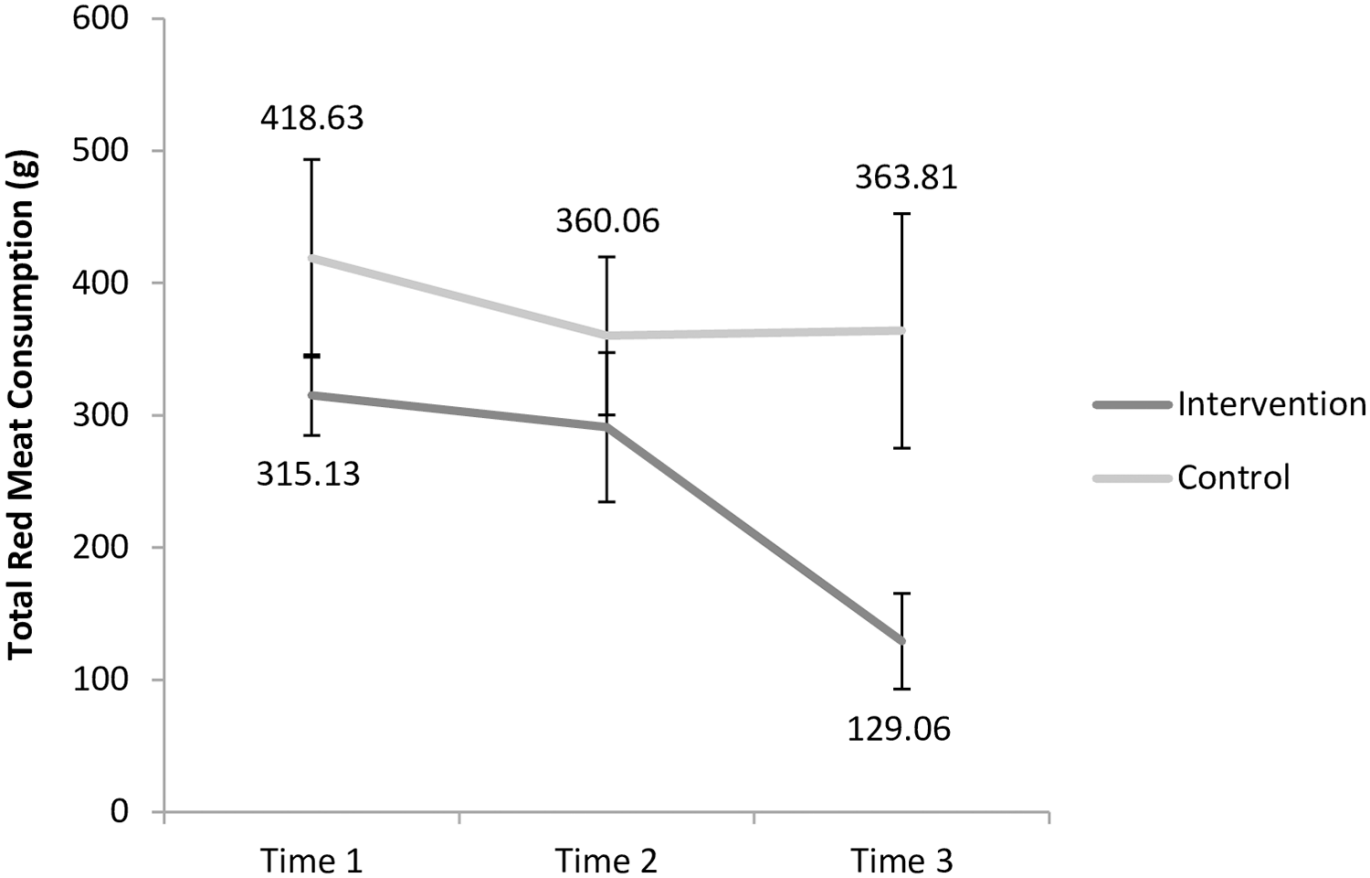
Changes in total red meat consumption over time in the control and intervention groups. Error bars are based on the standard error of the mean.

The ANOVA conducted on **total red meat consumption** also revealed an effect size above the η^2^_p_ = 0.04 threshold for the interaction between time and experimental condition. The pairwise comparisons conducted to interpret this interaction revealed that participants in the intervention group consumed significantly less red meat in total at Time 3 than at Time 1 (*M*_diff_ = 186.06, *p*<0.025). There was no difference in total red meat consumption between Time 1 and Time 2 (*M*_diff_ = 24.13, *p* = 0.705). In the control group, the total red meat consumed did not differ across these time points (all *p*s>0.025). Fig 2 illustrates this interaction. The main effects for Time and experimental condition observed in this analysis, and which both exceeded the η^2^_p_ = 0.04 threshold, should also be interpreted in light of the interaction.

The ANOVA conducted on **total white meat consumption** revealed a main effect of time that exceeded the η^2^_p_ = 0.04 threshold. However, pairwise comparisons conducted to interpret this effect showed that consumption of white meat remained the same, both from Time 1 to Time 2 (*M*_diff_ = 61.91, *p* = 0.211) and from Time 1 to Time 3 (*M*_diff_ = 86.34, *p* = 0.042).

The ANOVA next conducted on **red meat consumed during the week** revealed main effects for both time and condition for exceeded the η^2^_p_ = 0.04 threshold. Pairwise comparisons conducted to interpret the main effect of Time revealed that participants reduced their consumption of red meat during the week from Time 1 to Time 3 (*M*_diff_ = 73.91, *p*<0.025), but not from Time 1 to Time 2 (*M*_diff_ = 8.28, *p* = 0.828). Moreover, participants in the intervention group consumed less red meat during the week across all three time points (*M* = 149.38, *SD* = 82.20) compared to participants in the control group (*M* = 223.40, *SD* = 171.68).

The ANOVA conducted on **white meat consumed during the week** revealed an effect size above the η^2^_p_ = 0.04 threshold for the interaction between time and experimental condition. However, pairwise comparisons conducted within each of the two conditions showed that none of these comparisons achieved statistical significance (all *p*s>0.025).

In the ANOVA conducted on **red meat consumed on the weekend**, the main effects of time and condition as well as the interaction between time and condition all exceeded the η^2^_p_ = 0.04 threshold. Again, we focused on interpreting only the interaction. Pairwise comparisons conducted to interpret this interaction revealed that participants in the control group consumed less red meat on the weekend at Time 2 than at Time 1 (*M*_diff_ = 96.67, *p* = 0.018), although the difference between Time 1 and Time 3 was not significant (*M*_diff_ = 18.94, *p* = 0.681). In the intervention group, none of the comparisons of interest achieved the significance level of 0.025.

In the ANOVA conducted on **white meat consumed on the weekend** revealed a main effect for time that exceeded the η^2^_p_ = 0.04 threshold. However, pairwise comparisons conducted to interpret this main effect showed that participants in both conditions did not change their consumption of white meat on the weekend from Time 1 to Time 2 (*M*_diff_ = 42.72, *p* = 0.143) or from Time 1 to Time 3 (*M*_diff_ = 38.94, *p* = 0.175).

In the ANOVA conducted on **fish consumed on the weekend**, revealed an interaction between time and experimental condition that also exceeded the η^2^_p_ = 0.04 threshold. However, pairwise comparisons showed that none of the individual comparisons achieved the significance level (all *p*s>0.025).

Finally, in the ANOVA conducted on **consumption of cold cuts on the weekend** revealed that the interaction between time and condition also exceeded the η^2^_p_ = 0.04 threshold. Pairwise comparisons conducted to interpret this interaction within each group showed that none of these comparisons achieved statistical significance (all *p*s>0.025).

### Additional change analyses

Given the importance of reducing red meat consumption [55], and to capture the extent of the changes observed on that specific dependent variable (from Time 1 to Time 2 and from Time 1 to Time 3), we next conducted one-way ANOVAs with experimental condition as a between-participant factor and percent change of red meat consumed (in grams) as the dependent variable. Two scores representing percent change were calculated and included as dependent variables in these analyses: one score represented the difference between red meat consumed at Time 1 and Time 2, divided by red meat consumed at Time 1 (T1 to T2 change). The other change score represented the difference between red meat consumed at Time 1 and Time 3, divided by red meat consumed at Time 1 (T1 to T3 change). Such difference scores were computed for each participant individually and then averaged to represent the mean percent change in each condition. These additional analyses comparing the percent change of red meat consumption between the groups hence allow us to: quantify the changes taking place in each group, account for the baseline levels of red meat consumption, as well as examine if these changes are significantly different from zero in each group.

Results indicate that the change in red meat consumed from Time 1 to Time 2 was not significant (*F*(1,30) = 0.59, *p* = 0.448, η^2^_p_ = 0.02). However, the change in red meat consumed from Time 1 to Time 3 was significantly different in the intervention and control conditions (*F*(1,30) = 4.81, *p* = 0.036, η^2^_p_ = 0.14): Specifically, while participants in the intervention condition reduced their red meat consumption by 55.11%, those in the control condition increased their consumption of red meat by 6.14%. To further examine whether the change in red meat consumption was significantly different from zero in each group, one-sample *t*-tests conducted in each condition confirmed that the 55.11% decrease in red meat consumed in the intervention condition was significantly different from zero (*t*(15) = -4.25, *p* = 0.001), while the 6.14% increase in the control condition was not (*t*(15) = 0.25, *p* = 0.807).

### Psychological aspects of meat-eating

Then, a series of one-way ANOVAs were conducted to test if the intervention also had an impact on the attitudinal and emotional variables assessed at the end of the study (Time 3). As can be seen in Table 3, results of these ANOVAs revealed that participants in the intervention condition experienced less positive emotions toward meat-eating and greater inclusion of animals in the self compared to participants in the control condition.

**Table 3:** Comparison of the psychological variables across the conditions at Time 3.

Variable	Intervention Condition (*n* = 16)		Control Condition (*n* = 16)		*F*	*η*^2^_p_
	*M*	*SD*	*M*	*SD*		
Self-Determined Motivation (Index)	8.13	4.87	8.31	4.55	0.01	0.00
Ambivalence	3.52	1.66	4.10	1.84	0.89	0.03
Positive Emotions	2.74	0.76	3.48	1.19	4.39*	0.13
Negative Emotions	1.61	0.64	1.80	0.71	0.62	0.02
Intraindividual Conflict	3.33	1.60	3.04	1.44	0.29	0.01
Inclusion of Animals in the Self	4.88	1.41	4.25	1.29	1.71	0.05

*p<0.05.

Using a multiple linear regression with the entry method, in which we forced the predictors of interest into the model, we then examined which variables are the most important predictors of the change in red meat consumption from Time 1 to Time 3. All predictors were added in the model simultaneously to assess their relative contribution. In addition to the psychological variables (i.e., positive emotions, negative emotions, conflict, ambivalence, self-determined motivation, and inclusion of animals in the self) that were included as independent variables, we also included relevant demographic variables, which have known links to dietary behaviors. These relevant variables included: the number of hours of exercise per week, the number of hours spent at home per week, and the number of work hours per week, socio-economic status, and the BMI [72–76]. The overall model explained a significant amount of variance in the change in red meat consumption (*R*^2^ = 0.73, *p* = 0.042). That is, there was a marginal association between the work hours and change of red meat consumption, such that the more hours the participants worked during the week, the more they tended to increase their red meat consumption (*β* = 0.37, *p* = 0.057). Positive emotions also tended to predict an increase in red meat consumption (*β* = 0.45, *p* = 0.062). Finally, self-determined motivation to eat meat predicted a decrease in red meat consumption (*β* = -0.75, *p* = 0.006).

**Fig 3:**
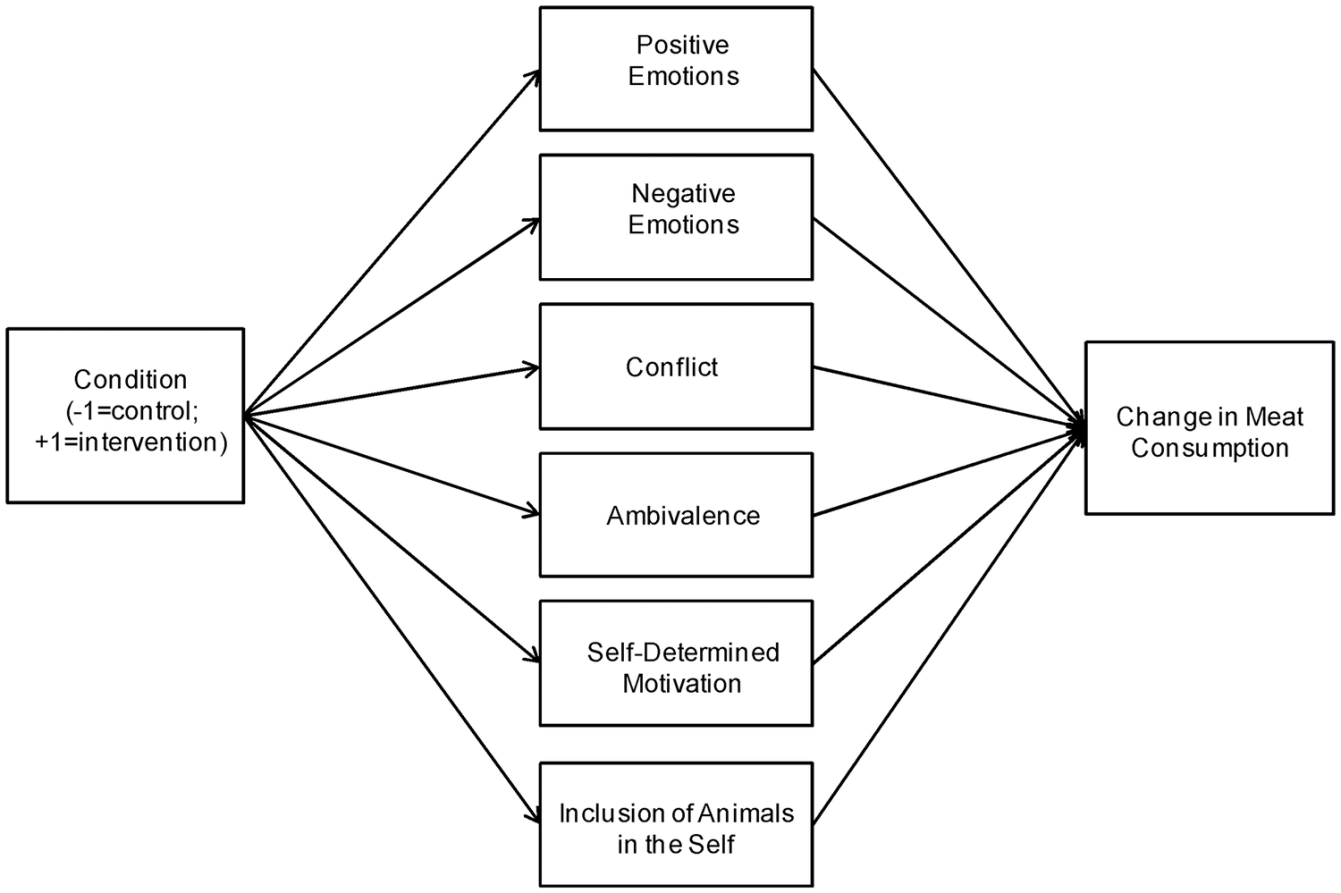
The proposed mediation model predicting a change in total red meat consumption from condition through the attitudinal and emotional variables.

To specifically test the mediating role of the psychological variables in the association between the conditions (i.e., experimental vs. control) and changes in meat consumption (Fig 3), we conducted mediation analyses using the PROCESS macro for SPSS [77]. While social psychological theories typically conceptualise attitudinal variables as mediators in the association between social (including normative) factors and behaviors (e.g., [19]), the emotional variables were included as mediators as well, for the sake of completeness, and given their important role in meat-eating [21]. Model 4 of the PROCESS macro allows testing the indirect effect of the predictor on the outcome through several mediators simultaneously. In this model, a bootstrap analysis is run to estimate confidence intervals around the relative indirect effect and determine its significance. The present analyses were based on 95% percentile bootstrap intervals with 5000 bootstrap samples. In addition, heteroscedasticity-consistent inference was not specified. As illustrated in Fig 3, condition (intervention vs. control) was entered as an independent variable, the psychological variables (i.e., positive emotions, negative emotions, conflict, ambivalence, self-determined motivation, and inclusion of animals in the self) were entered as mediators, and the difference in total red meat consumption between Time 1 and Time 3 was entered as a dependent variable.

There was an indirect effect of the condition on the change in red meat consumed through positive emotions (IE = -15.00, SE = 8.90, 95% CI [-34.93,-0.11]). Being in the intervention group (compared to the control group) predicted lower positive emotions about meat (*b* = -0.37, *p* = 0.045). Positive emotions, in turn, were associated with an increase in the red meat consumed (*b* = 40.61, *p* = 0.023). The direct effect of the condition on the change in red meat consumption was not significant (*b* = -17.67, *p* = 0.256), as well as the indirect effects of: negative emotions (IE = -1.85, SE = 5.68, 95% CI [-18.12,3.93]), conflict (IE = 1.74, SE = 5.86, 95% CI [-8.51,21.64]), ambivalence (IE = 4.51, SE = 7.16, 95% CI [-7.12,21.64]), self-determined motivation (IE = 0.74, SE = 6.98, 95% CI [-41.62,10.03]), and inclusion of animals in the self (IE = -3.11, SE = 4.34, 95% CI [-13.63,3.49]).

### Participants’ goals and the efficiency of the intervention and its components

Immediately after the in-lab presentation (at Step 2), participants in the intervention condition were asked to report their goals regarding meat consumption for the upcoming month. Out of the 16 participants, 15 (93.8%) reported that they intended to decrease meat consumption and 1 (6.3%) reported his intention to maintain current meat consumption. When asked more specifically about their plans to modify their meat consumption, 12 participants (75%) reported the intention to eat 1–3 less portions of meat per week and 1 participant (6.3%) reported the intention to avoid eating meat altogether. Three participants chose the ‘other’ option.

Upon completion of the intervention (at Step 6), 9 participants (56.3%) then reported having achieved the meat consumption goal they had set before the intervention, while 7 participants (43.8%) did not achieve their goal. In addition, at the end of the study, 5 participants (31.3%) classified themselves as flexitarians (eating meat occasionally), and the remaining 11 participants (68.8%) classified themselves as still being omnivores. This percentage of flexitarians at the end of the study is notable, as 100% of our participants at the start of the study self-identified as omnivores. None of the participants reported being vegetarian or vegan.

To gain insight into the intervention components that were the most effective for participants in the intervention group, we asked them to identify all of the components that they considered to have had an impact and to also select the one component that was the most important in helping them decrease their meat consumption. As seen in Table 4, the component which was selected most often was education about the harmful effects of meat-eating on human health, animal welfare, and the environment.

**Table 4:** Selection of the most effective components of the intervention.

	Selected as the Most Important Component (%)	Selected as an Effective Component (%)
**Education**	50	75
**Video**	18.8	50
**Self-Monitoring**	12.5	43.8
**Text Messages**	12.5	37.5
**Mind Attribution Manipulation**	0	0
**Other**	6.3	15.63

## Discussion

The current randomised controlled trial study aimed to test the impact of a novel and integrative multicomponent intervention that seeks to reduce meat consumption among young men over a one-month period. Building on research in social and health psychology, the intervention was composed of five components expected to decrease meat-eating and change people’s attitudes and emotions toward meat and animals. The novelties of the current study compared to prior studies that had aimed to reduce meat consumption (e.g., [55]) were: its use of a longer (4-week) randomised controlled trial design; the precise assessment of meat consumption, of different types; and its integration of different theoretical perspectives in social and health psychology (i.e., social norms, autonomy support, fear appeal, self-monitoring/goal setting; mind attribution to meat-animals) to build the intervention. Doing so allowed us to capture the psychological processes that underpin the changes in meat consumption over time.

Results confirmed the effectiveness of the intervention in producing a greater decrease in meat consumption over time. Specifically, the consumption of red meat decreased significantly from Time 1 to Time 3 in the intervention group, but not from Time 1 to Time 2. The fact that we observed significant drops in meat consumptions specifically from Time 1 to Time 3 suggests that some time may be required to implement this change into one’s life habits. Furthermore, this finding is encouraging given at Time 3, no more text messaging were sent to participants, suggesting that the impact of the intervention went beyond the duration of this specific component of the intervention (cf. [55]).

Interestingly, the reduction in red meat consumption appears to be driven most by a decrease in this type of meat eaten during the weekend per se (as attested by the effect sizes and the observed means). The fact that these effects emerged particularly strongly on the weekend suggests that dietary behavior may be more amenable to change on the weekend then on week days. The mechanism that could explain this phenomenon is presently unclear. However, since unhealthier eating habits are observed during the weekend [78–79], it may have been easier for the participants in the present study to reduce their meat consumption habits during the weekend than the week days. Finally, and to further illustrate the extent of the change observed in red meat consumption per se, we found that participants in the intervention condition decreased their red meat consumed by 55.11% from baseline (Time 1) to four weeks later (Time 3)–which represented a significant decrease–, whereas participants in the control condition (non significantly) increased their consumption by 6.14% during this period of time.

As an additional indicator for the effectiveness of the current intervention, we found that nearly all (15 of the 16) of the participants in the intervention group reported (at Time 1) that they had intended to decrease meat consumption during the upcoming month. Interestingly, upon completion of the intervention (at Time 3), a majority of participants in this condition reported having achieved the meat consumption goal they had set before the intervention, and approximately a third of the participants even self-identified themselves as flexitarians. It should be noted that the changes in meat consumption in the intervention group may have been impacted most by the education component of the intervention as this component was identified to be the most important by participants. Collectively, results of the present study suggest that the multi-component intervention used may be a promising strategy to decrease meat consumption. Accordingly, healthcare professionals (e.g. psychologists, nutritionists) could use this type of intervention during the planning of their intervention programs since red meat and cold cuts consumption is associated with an increased risk of developing cancer [80].

In terms of the psychological variables, only positive emotions toward eating meat were found to be (negatively) impacted by the intervention. This psychological variable was also the only significant mediator in the association between the condition participants were in and their reduction in meat consumption over the course of the study. This result suggests a particularly clear role for the affective processes involved in meat-eating and aligns with prior work showing the importance of affective (compared to cognitive) processes in the intention to reduce meat-eating (e.g., [67]). Positive emotions derived from eating meat is also an important justification for why people report to eat meat [81], and in this sense is likely to promote the maintenance of this behavior over time. Reduction in positive emotions toward meat-eating, as was the case in our intervention, was associated with a decrease in this dietary behavior, possibly due to the belief that it becomes less legitimate.

In the multiple regression that included the demographic and psychological variables, positive emotions were again a significant negative predictor of the change in red meat consumption from Time 1 to Time 3, along with self-determination to eat meat. This latter finding suggests that as people reflectively choose to eat meat and have integrated this habit into their life in an autonomous manner, the more this conscious decision then predicts a *lower* tendency to eat meat. This finding aligns with prior work that has investigated the regulation of eating behaviors; in this work as well, being mindful and self-determined–rather than pressured and coerced by others–also predicted more healthy eating behaviors [82].

### Limitations and future directions

Although the current focus on young men allows, methodologically, to ensure a homogeneous sample yet also provides a stringent test to the current intervention (e.g., [83]), further research will need to be conducted among larger and more heterogeneous samples. Such samples should include participants of both genders, and of different ages, socioeconomic, and ethnic/cultural groups, as these demographic factors have been found to come into play in meat consumption [57, 84]. Doing so will ensure the generalizability of the current findings. In addition, while conducting the current study over a one-month period represents an improvement relative to prior studies on meat-eating, which were typically conducted over shorter time-frames, future work should include follow-up assessments that take place over several months and beyond [85]. The replication of the current results among more diverse populations and also over longer timeframes will not only ensure the generalizability of the current findings, but also pave the way for a precise cost-effectiveness analysis of the current intervention. Furthermore, the psychological variables that were tested as mediators should ideally be assessed *prior* to the moment at which participants reported their final levels of meat consumption. Doing so will allow to assign a clear role to each variable, in line with their conceptualisation and with prior work on attitudes [19]. Finally, while the current intervention included multiple components, of which the educational and fear appeal (video) appeared most effective to participants, future work could unpack each of these components and test their respective impacts.

### Conclusion

Results indicate that the multi-component intervention was effective in significantly reducing meat consumption in young men. Our results may be useful for clinical and practical purposes. It is important to educate health care professionals regarding the potential beneficial effects of this novel multi-component intervention. Specifically, methods such as self-monitoring and goal setting as well as fear appeal could be implemented in future interventions in order to promote meat reduction among young men, and possibly additional populations as well, which could lead to the improvement of several health outcomes (e.g. cancer, diabetes and cardiovascular diseases). Whereas these findings should be considered preliminary, they may stimulate additional research on the impact of such novel and integrative interventions on both health and environmental markers.
